# Effectors and Effector Delivery in *Magnaporthe oryzae*

**DOI:** 10.1371/journal.ppat.1003826

**Published:** 2014-01-02

**Authors:** Shijie Zhang, Jin-Rong Xu

**Affiliations:** 1 NWAFU-Purdue Joint Research Center, College of Plant Protection, Northwest A&F University, Shaanxi, People's Republic of China; 2 Department of Botany and Plant Pathology, Purdue University, West Lafayette, Indiana, United States of America; Duke University Medical Center, United States of America

Rice blast caused by *Magnaporthe oryzae* is one of the most destructive fungal diseases of rice and a model for studying fungal-plant interactions. The fungus penetrates plant cells with appressoria and develops the narrow primary invasive hyphae (IH) and, subsequently, the bulbous secondary IH. As a hemibiotrophic pathogen, biotrophic IH are enclosed in the extra-invasive-hyphal membrane (EIHM) produced by the plant cells [1]. Like many other fungal pathogens [2], *M. oryzae* secretes effector proteins to manipulate plant immunity and physiology to promote infection.

## AVR Effectors

Avr proteins, a special class of effectors encoded by the avirulence (*AVR*) genes, could be recognized by corresponding R proteins and lead to the race-specific recognition [3]. In the rice–*M. oryzae* interaction, over 40 *AVR* genes have been identified. Among the *AVR* genes that have been cloned, all but *ACE1* encode secreted proteins expressed in IH. *ACE1* is specifically expressed in appressoria, and it encodes an intracellular hybrid PKS-NRPS protein [4]. Avr-Piz-t and Avr-Pita are the other two Avr proteins with known biochemical functions. Avr-Piz-t functions to suppress pathogen-associated molecular pattern (PAMP)-triggered immunity by inhibiting the ubiquitin ligase activity of the rice RING E3 ubiquitin ligase APIP6 [5]. *AVR-Pita* encodes a putative neutral zinc metalloprotease [6] and it belongs to a gene family with at least two additional members [7].

*PWL1*, *PWL2*, *PWL3*, and *PWL4* are members of another *AVR* gene family [8]. Pwl effectors are small, glycine-rich proteins that are present in rice pathogens and function as avirulence proteins in infection of weeping lovegrass and finger millet. In contrast, *AVR1*-CO39 was cloned from a weeping lovegrass isolate. Its specific expression in IH triggers hypersensitive response (HR) and resistance in cultivars carrying the Pi-CO39 R gene [9]. Avr-Pia, Avr-Pik/km/kp, and Avr-Pii were identified in the same re-sequencing study of strain Ina168 [10]. Avr-Pia directly interacts with Rga5-A, which also interacts with Avr-CO39 [11].

## Non-AVR Effectors

The best characterized non-Avr effector in *M. oryzae* is Slp1, a secreted LysM protein that is accumulated at the interface between IH and EIHM [12]. Slp1 is dispensable for appressorium penetration but required for invasive growth *in planta*. It competes with the chitin elicitor binding protein CEBiP for binding to chitin oligosaccharides. Thus, Slp1 functions to suppress chitin-induced plant immune responses, including generation of reactive oxygen species and expression of defense-related genes [12].

Using an expression profiling approach, a number of low molecular weight secreted proteins specifically expressed or highly induced in biotrophic invasive hyphae were identified, including 58 candidate effectors that were up-regulated over 10-fold during plant infection. Four of them, *BAS1–BAS4*, were confirmed to be fungal biotrophy-associated secreted (BAS) proteins [13]. MC69 was identified by systematic disruption of *in planta* expressed secreted protein genes [14]. The 54-aa Mc69 protein was essential for IH development, although it was not translocated into rice cytoplasm. In a separate study, five secreted protein genes named *MoCDIP1–5* were found to induce plant cell death in a transient expression assay with rice protoplasts. Four of them also induced cell death in *Nicotiana benthamiana* [15].

## Localization of Fungal Effectors in Plant Cells

**Figure 1:**
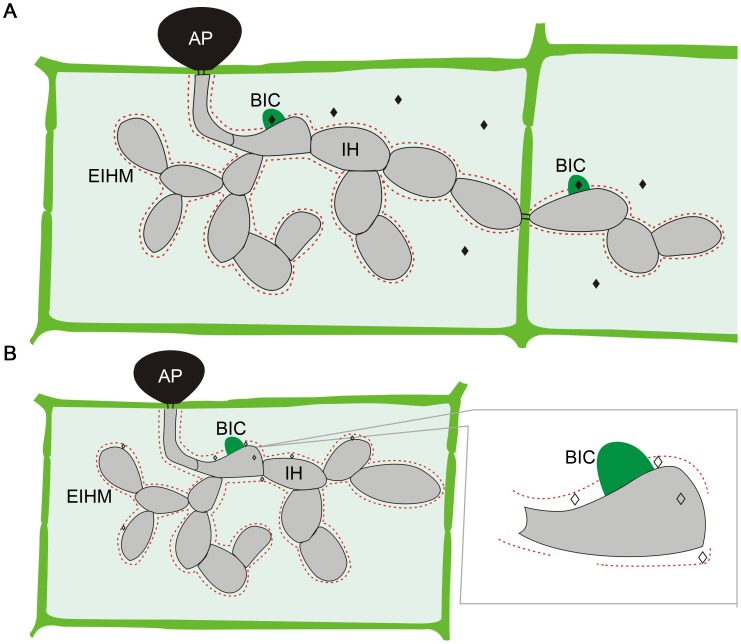
Localization of *M. oryzae* effectors during plant infection. **A.** Cytoplasmic effectors (♦) are secreted into the biotrophic interfacial complex (BIC) before being translocated into plant cells. **B.** Apoplastic effectors (◊) are secreted into the space between the fungal cell wall and extra-invasive-hyphal membrane (EIHM). Like IH, effector proteins may move from cell to cell via plasmodesmata. AP, appressorium.

*M. oryzae* effectors can be divided into two distinct types based on their localization in plant cells (Figure 1) [16]. Cytoplasmic effectors, including Avr-Pita, Pwl1, Pwl2, Bas2, and Avr-Piz-T, are preferentially accumulated in the biotrophic interfacial complex (BIC) before being delivered into plant cells. The BIC is a distinct plant-derived, membrane-rich structure developed at the tip of primary IH by *M. oryzae*. In each newly invaded rice cell, effectors are first secreted into BICs before delivery. The BICs are persistent and left behind when the primary IH differentiates into the secondary IH. In addition, the fungus continues to secrete effectors into BICs even after IH have grown extensively as pseudohyphae and invaded neighboring plant cells [17].

On the other hand, apoplastic effectors such as Bas4, Avr1-CO39, and Slp1 are not associated with the BIC. After secretion, they are dispersed in the extracellular space between the fungal cell wall and EIHM. In the rice cells colonized by transformants expressing the Bas4-GFP fusion protein, GFP signals appeared to outline the IH [13], consistent with its extracellular localization. To date, it is not clear how the apoplastic effectors are recognized by surface receptors or translocated into plant cells to interact with their intracellular targets. Furthermore, no specific protein motifs or sequences have been identified in the cytoplasmic or apoplastic effectors that are responsible for their localization in plant cells after being secreted. Therefore, it is impossible to predict whether an effector is apoplastic or cytoplasmic solely based on its amino acid sequence.

## Effector Secretion Systems

**Figure 2:**
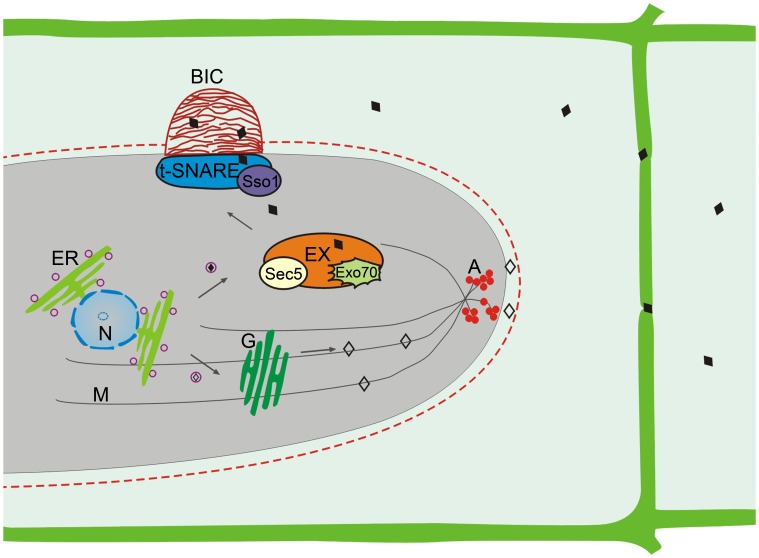
Two effector secretion mechanisms identified in *M. oryzae*. The apoplastic effectors (◊) utilize the conventional ER-Golgi secretion pathway for secretion. Disruption of the cytoskeleton by benzimidazoleand LatA treatments interferes with the secretion of apoplastic effectors. In contract, the secretion of cytoplasmic effectors (♦) into the BIC is independent of the Golgi bodies and cytoskeleton but involves the exocyst complex and t-SNAREs. A, cortical actin; ER, endoplasmic reticulum; EX, exocyst complex; G, Golgi bodies; M, microtubules; N, Nucleus.

Consistent with two types of effectors, two distinct effector secretion systems have been identified in *M. oryzae* (Figure 2) [16]. Cytoplasmic effectors are delivered into plant cells via the BIC, which is independent of the Golgi-dependent secretory system. Instead, secretion into the BIC is associated with a novel form of secretion involving the exocyst complex and t-SNAREs. Targeted deletions of the exocyst components *SEC5* and *EXO70* resulted in impaired secretion of cytoplasmic effectors and pathogenicity defects but had no effect on the secretion of apoplastic effectors [16]. Mutants deleted of the t-SNARE component *SSO1* were defective in BIC development and pathogenesis [16].

The apoplastic effectors are secreted from IH by the conserved ER to Golgi secretory pathway that is independent of the BIC. Treatment with Brefeldin A that interferes with Golgi-dependent secretion inhibited the secretion of apoplastic effectors such as Bas4 and Slp1 but had no effect on the localization of cytoplasmic effectors Pwl2, Bas1, and Bas107 to the BIC [16]. Furthermore, actin and microtubules essential for vesicle trafficking are required only for the secretion of apoplastic effectors but not for cytoplasmic effectors. Many genes involved in the ER to Golgi secretory pathway and post-translational modifications also are likely important for the secretion of apoplastic effectors in *M. oryzae*. For example, the *LHS1* ER chaperone gene is required for the translocation and secretion of apoplastic effectors such as Slp1 [18].

## Translocation of Effectors into Plant Cells

Several cytoplasmic effectors, including Pwl2, Bas1, and Avr-Piz-t, are translocated into the cytoplasm of rice cells [5], [13]. Whereas some apoplastic effectors may be recognized by host plant surface receptors, the others that have intracellular targets, such as Avr1-CO39, also enter plant cells. In rice leaf sheath cells penetrated by fungal transformants expressing the Pwl2 and Avr-Piz-t proteins tagged with NLS and mRFP sequences, red fluorescence was observed in the nucleus of plant cells [17]. However, the underlying molecular mechanism responsible for effector translocation into plant cells is not clear. In experiments with purified recombinant proteins, the native Avr1-CO39 protein was translocated into rice protoplasts, indicating that it can enter plant cells independent of fungal factors [19]. In Oomycete pathogens such as *Phytophthora* species, the RXLR sequence is important for the translocation of effector proteins into plant cells [20]. Nevertheless, there is no evidence for the presence of functional RXLR sequences in *M. oryzae* effector proteins.

## Movement of Effectors in Plant Cells

In the rice leaf sheath cells penetrated by *M. oryzae*, the cytoplasmic effectors Pwl2 and Bas1, but not apoplastic effector Bas4, were moved to neighboring cells ahead of the invasive hyphae [17]. Red fluorescence was observed in the nucleus of rice leaf sheath cells penetrated by a transformant expressing the *PWL2*-mCherry-NLS fusion construct and a number of surrounding cells without IH. Fungal effectors entering un-colonized plant cells may be able to suppress host defense responses or elicit susceptibility. In fact, it takes a shorter time (2 h) for IH to move through the subsequently invaded cells than in the first colonized cells (12 h) [17].

Interestingly, movement of cytoplasmic effectors from vein-associated cells into neighboring cells was rare and of low efficiency in comparison with effector movement among regular epidermal cells [17], suggesting that effector trafficking depends on rice cell types. In addition, unlike the 39.3 kD Pwl2-mCherry fusion, the 68.3 kD Pwl2-tdTomato protein was defective in the movement from the penetrated cells to surrounding cells [17]. Therefore, cell-to-cell effector translocation also depends on the protein size. These results indicate that *Magnaporthe* effectors may be moved symplastically through plasmodesmata [17]. The movement of effector proteins and invasion of neighboring cells by IH may be co-regulated because IH of *M. oryzae* preferentially contacted and crossed the plant cell wall at the pit fields [1].

Although more and more fungal effectors are being discovered, our understanding of effector delivery and cell-to-cell movement *in planta* is relatively limited in comparison with that of bacterial effectors. Studies in *M. oryzae* showed that some effectors may be secreted via an unconventional protein secretion system to the BIC [16]. The BIC-like structures may also exist in other plant pathogenic fungi, particularly other hemibiotrophic pathogens. To date, no common plant entry sequence has been identified in fungal effectors, indicating that fungi may utilize a variety of mechanisms for effector translocation. *M. oryzae* and other fungal pathogens may have conserved mechanisms to recognize plasmodesmata for the movement of effector proteins and spreading of IH.
